# Cognitive and Field Testing of a New Set of Medication Adherence Self-Report Items for HIV Care

**DOI:** 10.1007/s10461-013-0610-1

**Published:** 2013-09-28

**Authors:** Ira B. Wilson, Floyd J. Fowler, Carol A. Cosenza, Joanne Michaud, Judy Bentkover, Aadia Rana, Laura Kogelman, William H. Rogers

**Affiliations:** 1 Department of Health Services, Policy and Practice, Brown University School of Public Health, G-121-7, 121 South Main St., Providence, RI 02912 USA; 2 Center for Survey Research, UMass Boston, Boston, MA USA; 3 Department of Infectious Diseases, Miriam Hospital, Providence, RI USA; 4 Department of Infectious Diseases, Tufts Medical Center, Boston, MA USA; 5 The Health Institute, Tufts Medical Center, Boston, MA USA

**Keywords:** HIV, Medication adherence, Self-report, Questionnaires, Survey methodology

## Abstract

We conducted four rounds of cognitive testing of self-report items that included 66 sociodemographically diverse participants, then field tested the three best items from the cognitive testing in a clinic waiting room (*N* = 351) and in an online social networking site for men who have sex with men (*N* = 6,485). As part of the online survey we conducted a randomized assessment of two versions of the adherence questionnaire—one which asked about adherence to a specific antiretroviral medication, and a second which asked about adherence to their “HIV medicines” as a group. Participants were better able to respond using adjectival and adverbial scales than visual analogue or percent items. The internal consistency reliability of the three item adherence scale was 0.89. Mean scores for the two different versions of the online survey were similar (91.0 vs. 90.2, *p* < 0.05), suggesting that it is not necessary, in general, to ask about individual medications in an antiretroviral therapy regimen when attempting to describe overall adherence.

## Introduction

While a wide variety of self-report measures have been developed to assess adherence with HIV ART, few of the questionnaire items that make up these measures have been subjected to rigorous cognitive testing to ensure that the items are consistently understood by respondents. Accurate self-reports of medication could be useful in routine clinical care because research has consistently shown that physicians’ assessment of their patients’ adherence with ART is inaccurate [1–4]. They could also be useful for research when more objective measures such as MEMS caps [5] or unannounced pill counts [6, 7] are impractical or too costly [8, 9].

A number of self-report measures of medication adherence have been developed for chronic medical conditions such as hypertension and diabetes (e.g., Morisky), with different levels of validity testing [10–13]. For HIV, a wider variety of instruments have been developed and used [14].The validity of these instruments has been assessed, in general, by examining their relationship to laboratory outcomes, most commonly viral loads. Correlations with viral loads are consistently in the 0.3–0.4 range [14, 15], and sometimes a little better. Previous work by our group showed that a rating item performed better than either a frequency item or a percent item using electronic drug monitoring (MEMS) as a gold standard [16]. Subsequent work by others has confirmed this finding [17, 18]. However, little is known about why certain items appear to perform better than others [15], or whether further improvements can be made.

Another important issue for survey designers is whether it is necessary to ask about each of the individual medications that make up an antiretroviral therapy regimen, or whether one can ask about the regimen in the aggregate. Relatively few papers have attempted to assess differential adherence [19–23]. While some of these studies suggest that it is not necessary to measure individual medications [19, 20, 23], these were relatively small, single site studies, and other studies suggest that differential adherence may be consequential [21, 22]. Thus it remains unclear whether the extra effort needed to measure adherence with each component of a regimen, which in the case of a three-drug regimen triples the respondent burden, is worthwhile.

To better understand why some items perform better than others, and to try to optimize the quality and performance of such measures, we conducted an extensive, iterative series of in-depth cognitive interviews with a socioeconomically and demographically diverse group of patients with HIV in Massachusetts and Rhode Island to find out how they understood the survey items. We then conducted pilot tests of the best items in over 350 patients who completed a pencil-and-paper version of the survey, and over 6,400 patients who completed an online version of the survey. The online version included a randomized test of whether responses differed if respondents focused on an individual medication or the antiretroviral regimen as a whole. We had three specific study questions: (1) Which item stems were most consistently understood by respondents and which response tasks could respondents use best to provide answers? (2) Can patients respond accurately to questions about their whole ART regimen or is it necessary to ask questions about individual pills in the regimen? (3) What are the psychometric characteristics of the resulting adherence measurement scales?

## Methods

### Cognitive Testing

#### Purpose

Cognitive interviews allow researchers to: learn about respondents’ comprehension of candidate survey items; identify any unclear concepts, questions, or terms; and evaluate whether or not the answer provided accurately reflects what respondents have to report.

#### Participants

Subjects for the cognitive testing were recruited from the HIV clinics at two urban Academic Medical Centers in MA and RI. Eligible patients where those who were taking antiretroviral therapy, taking at least one other daily medication for a chronic condition, spoke English, and had at least one detectable HIV plasma viral load in the last two recorded tests. The criterion of taking at least one other daily medication for a chronic condition was so we could determine whether these items worked equally well for ART and medications for other conditions. Potential subjects were identified by signs in the exam rooms (self-referral), recommendations from treating physicians, and medical record reviews. They were paid $80 dollars for up to 2 h of their time. A total of 270 patients were screened, 81 proved to be eligible, and 66 completed an interview. Of the 15 who did not complete an interview, five did not show up for their appointments, two cancelled their appointments before the scheduled time, one decided not to participate after arriving for the appointment but before consenting, and seven did not return calls to schedule an interview. There were no significant differences (*p* < 0.05) between participants and non-participants in age and gender (the only available variables).

#### Questionnaires and Interview Process

##### Strategy

Our goals were to identify items and response categories that were relatively simple, and consistently understood by respondents from different socioeconomic backgrounds. Concepts we explored included being adherent for a specific period versus generally adherent; the kinds of specific tasks that respondents could understand and recall (taking, missing, ability to take, etc.); adherence (execution or implementation of a regimen) versus persistence (stopping altogether); comprehension of different response tasks (yes/no, visual analogue scales, numbers/percents vs. adjectives/adverbs) and the problems or biases that existed with each; and whether responses would be clinical meaningful or interpretable. In addition, although it is difficult, we tried to minimize phrasing the might increase social desirability pressures to overreport adherence such as “missing doses”. Finally, we also wanted to design an instrument that was simple enough to be self-administered.

##### Item selection

The questionnaire design process began by collecting as many instruments and items as we could that had attempted to measure self-reported medication adherence. We first conducted a literature review using combinations of the following search terms: HIV, highly active antiretroviral therapy, medication adherence, self-report, questionnaires, survey methodology. In addition we reviewed references of review articles and directly contacted a number of investigators. There were dozens of such questions that varied in their reference period, how they were worded, what respondents were supposed to report about, and the response tasks respondents were supposed to use to provide answers (a detailed list of the items we considered is available from the first author upon request). We focused our initial attention on the question wording and response tasks that seemed to be used most often and had the strongest face validity.

##### Cognitive testing protocol

We conducted four rounds of cognitive testing. In the first three rounds first asked the respondents to list the medicines they were taking. The instruments asked one series of questions about an HIV medication and another series about a medication they were to take daily for another chronic condition. This approach gave us an opportunity to use the cognitive interview to insure that the items worked similarly for other (non-antiretroviral) medications. The initial instrument we created asked various types of questions for 1 week and 1 month reference periods and included questions with several different response tasks. The instrument for the second round included some new concepts that had not been included in the first round. We also began to test slightly reworded questions that had been changed to address issues identified in the first round. The third round of questions consisted of what we considered to be the strongest candidates for a final instrument. A final fourth round, shorter than the previous instruments, consisted of a short list of questions that we believed would constitute our final instrument. The specific items tested in each of these rounds are available from the corresponding author upon request.

The cognitive interview consisted of two parts. First, respondents completed all of the questions in a self-administered paper form. The interviewer then went back over the questions, one by one and talked to the respondent about their responses. Interviewers used a semi-structured protocol that included a set of probes for each question that was designed to help understand how respondents understood questions and went about answering them. Before completing the discussion of each question, interviewers were instructed to use additional probes as needed to collect information about each of the four elements of question answering identified by Tourangeau: comprehension, retrieval, transformation, and providing an answer [24, 25]. Interviews were audio-taped, with respondent permission. Interviewers then could use the tape recording to help write up their observations about question issues after each interview had been completed. Interviewers were debriefed by investigators at the UMASS Center for Survey Research, and changes to the instrument were made based on the cognitive testing results.

##### Analyses

The results from the cognitive testing were used in two ways to evaluate the questions. First, interviewers reported on their experiences with each of the four elements of the questions answering process (comprehension, retrieval, transformation and answering) plus the use of the reference period for each question. Second, since a number of questions were asked that essentially addressed how well medications were taken, we looked at the consistency of answers across questions. One specific approach that we relied on most looked at the number of days in the past month respondents said they had missed taking any medications. Answers to other questions were tabulated by whether the respondent reported missing no medications or reported missing at least one. The more answers to other questions seemed inconsistent with the number of missed medications reported, the more concern there was about the reliability of those answers. After four rounds of testing, three items clearly emerged as the best items, and those were rotated forward into pilot testing.

### Field Testing

#### Participants

The three items that emerged from the cognitive testing were then pilot tested in two settings. A pencil and paper version of the survey was conducted in the same two clinics where the cognitive testing was done. Patients were invited to participate, given the short (two sides of a page) survey, and told where they could anonymously return it by depositing it in a locked box. All patients who had not participated in the cognitive testing and were using HIV antiretroviral medications were eligible.

Cross-sectional, national, internet-based surveys were administered to U.S. based members of one of the most popular American social networking site for gay and bisexual men and other men who have sex with men (MSM), administered by the OLB Research Institute at Online Buddies, Inc.

#### Questionnaires

The pencil-and-paper version of the questionnaire included an item asking how many different HIV medicines the subject takes, the three adherence questions, and items about how long ago the patient first started taking antiretrovirals, gender, age, education, ethnicity, and race. For each of the three items asking about antiretroviral medication adherence, this pencil-and-paper version of the questionnaire used the phrase their “HIV medicines” which we had determined in the cognitive testing was consistently understood as referring to HIV antiretroviral medications.

The web version of the survey was randomized into two versions which allowed us to test whether patients responded differently when asked about “your HIV medications” as a group compared with asking about individual HIV medications one at a time. Half of the patients who took the web version were shown a color chart of all HIV antiretroviral agents currently on the market in pill form, and then presented with an alphabetical list of all of the medications. These patients were then asked: “FROM THIS LIST, CHOOSE ONE OF THE HIV MEDICINES THAT YOU ARE TAKING.” A picture of the selected medication was shown for confirmation. The name of that medication was included in all subsequent items. For example, if a patient chose Atripla as their medication, an item on the survey would appear as follows: “Now think about the last 30 days. How would you rate how well you did taking your Atripla?” The other half of the respondents received a survey which asked about “your HIV medicines” as a group, in the same way the items appeared on the pencil and paper survey.

#### Analyses

We calculated descriptive statistics for the pencil and paper and both arms of the web survey. Item responses for the three adherence items were linearly transformed to a 0–100 scale [26, 27]. A summary of the individual adherence items was calculated as the mean of the three individual items. We assessed the internal reliability consistency of the resulting scale using Cronbach’s alpha. Differences between the two arms of the web survey and between the web survey in the pencil and paper survey were assessed by Chi square tests for dichotomous and categorical variables, and t-tests for continuous variables.

## Results

### Cognitive Testing

#### Descriptive Characteristics

Sixty-six individuals participated in the four rounds of cognitive testing. Of the 66, 70 % were male, median age was 51 years, 71 % had education of high school or less, 59 % were non-white, and 5 % were Hispanic.

#### Lessons Learned

The following are some of the key findings from the cognitive interviews (Table 1). Most subjects were unable to construct a medication list from memory. Pills versus non-pill medications (e.g., inhalers) also caused confusion, and some participants did not know how to report on pills taken “as needed.”

**Table 1:** Lessons learned from cognitive testing by item stem and response option

Lesson	Comment
Item stem	
Time frame	No consistent understanding of “the last week” or “the last month”; better the last 7 or 30 days
Attention to reference period	Attention to the reference period was poor overall; patients estimate rather than count
Taking “as prescribed”	Understood inconsistently
Understanding of “dose”	Understood consistently
Response option	
Visual analogue scales and percents	Both worked poorly
Use of the word “perfect”	Worked poorly
Options that express feelings	Worked poorly
Words vs. numbers	Subjects level of recall is more appropriate to verbal than numerical answers and subjects more comfortable with adjectives and adverbs than numbers as way of providing answers

One focus of testing was to learn about what the best “reference period” was and how to describe that reference period. There proved to be no consistent understanding of “the last week” or “the last month.” The phrase “the last week” was interpreted variably as the last 7 days, the previous Monday to Sunday interval, and the previous Sunday to Saturday interval. The same was true for “the last month.” Subjects had a much more consistent understanding of time periods when expressed as number of days, such as “the last 7 days” or “the last 30 days.” Despite this general understanding, overall, subjects’ attention to the reference period was poor. They tended to answer generally about the time period rather than focus on the exact time period. Also, as one would expect, ability to retrieve details was worse for 30 days than for 7 days. However, respondents found it difficult to recall precisely for either reference period.

We tested a series of questions that asked about taking medicine “exactly as the doctor prescribed.” Subjects found this difficult for several different reasons. One problem is that it is not always a physician that does the prescribing. Another problem was that doctors differ in the extent to which they describe how medicines should be taken, with some giving little instruction, and others giving more. However, even if a physician did provide instructions, patients often could not remember the details of the instructions. We tested several variations to solve this problem, and patients most consistently understood the questions that used the phrase “the way you are supposed to take” your medicine.

We found that subjects usually had a consistent understanding of the concept of a “dose.” However, there was inconsistent application of this concept when answering the actual survey questions, particularly in cases where there was twice a day dosing.

With regard to response options, both visual analogue scales and asking about percents worked relatively poorly. Most people have to do some math to respond to these questions, and they often make errors when they do. Moreover, there are some subjects who made a guess or an estimate without doing any math. In general, we found that participants were not consistently able to understand and apply fractions or percentages.

A scale that ranged from “very poor” to “perfect” did not work well. Some subjects answered by saying that “no one is perfect.” Many refused to endorse “perfect” even when their adherence was 100 % on other scales. There was also confusion about a scale we tested that asked “Overall, how do you feel about the way you took [medication name] in the last 7 days?” Response options ranged from delighted to terrible. This caused confusion between how they felt physically and how they took their medications. Also, “delighted” was not a term that many associated with medication taking.

In general, subjects were more comfortable and confident using adjectives and adverbs as response options than they were with quantitative assessments. One partial reason is that, as we noted above, detailed recall was far from perfect. The level of detail respondents could recall was more appropriate for verbal than quantitative answers. For those with less than perfect adherence, as previously noted, subjects tended to estimate rather than count or enumerate, and words seem to map onto this cognitive estimation process more accurately and more comfortably for most patients than numbers.

The three best performing items are shown in the Appendix.

### Pilot Testing of the Three Best Performing Items

#### Descriptive Characteristics

Not surprisingly the web-based and clinic-based samples were different from each other (Table 2). The web-based sample was more male (<99 vs. 75 %), less Hispanic (9 vs. 19 %), less African-American (5 vs. 25 %), and better educated (88 % education beyond high school vs. 59 %).

**Table 2:** Participant characteristics

Characteristic	Web based		Paper
	One med (*N* = 3,231^a^)	All meds (*N* = 3,254^a^)	*N* = 351
Age [mean years (SD)]	46.7 (10.0)	47.2 (10.0)	49.4 (9.7)
Gender (% male)	99.3	99.4	75.1
Hispanic (%)	9.5	8.9	19.0
Race (%)^b^			
White	89.5	89.1	57.0
African American	4.7	5.1	25.1
Asian	1.2	1.2	1.7
Pacific Islander	0.4	0.3	0.3
Native American	1.8	1.6	4
Other	4.4	5.0	14.5
Education			
8th grade or less	0.2	0.2	4.0
Some high school but did not graduate	1.3	1.3	11.5
High school graduate or GED	9.8	10.5	25.0
Some college or 2-year degree	34.6	34.9	33.0
4-year college graduate	24.6	25.4	13.5
More than 4-year college degree	29.5	27.7	12.9

^a^Those who agreed to participate among *N* = 3724 (One med) and *N* = 3768 (all meds)

^b^Sum of the percent is >100 because of multiple responses

#### Inquiring About Whole ART Regimen Versus Individual Pills

Scores on each of the three items, and on the summary three-item scale, were similar between the randomized groups (Table 3). Regarding the randomized comparison, the adherence scale score for the single item scale was slightly higher than the adherence scale score for the item that asked about the whole regimen (91.0 vs. 90.2, *p* < 0.05). Though it was statistically significant, given the magnitude of this difference (0.8 points on a 100 scale), we did not consider this difference clinically important. The difference between the whole regimen arm of the web-based trial and the clinic sample was greater (90.2 vs. 88.8), but because clinic patients were not included in the randomization, direct comparisons are not appropriate. Given the sociodemographic characteristics of the clinic sample (more women, more persons of color, lower educational levels) one might anticipate lower adherence.

**Table 3:** Descriptive data on adherence items (0–100 scale, see Appendix for exact wording)

Item (mean (SD))	Web based		Paper
	One med (*N* = 3,231*)	All meds (*N* = 3,254*)	*N* = 351
How many days NOT missed…	95.8 (11.4)	95.1 (12.9)*	94.7 (14.1)
How good a job did you do…	87.8 (19.8)	86.8 (20.9)	84.2 (21.7)*
How often did you take…	89.7 (17.3)	88.8 (18.1)*	88.0 (19.9)
Mean of the three item scales	91.0 (14.6)	90.2 (15.8)*	88.8 (17.1)*
Cronbach’s alpha	0.86	0.89**	0.89*

Using One med as a reference, scales were compared by *t* test (Non parametric tests’ results were the same) and Cronbach’s alpha were compared by Fisher’s z-transformation

* *p* value <0.05, ** *p* value <0.01

#### Item and Scale Distributions

**Fig. 1:**
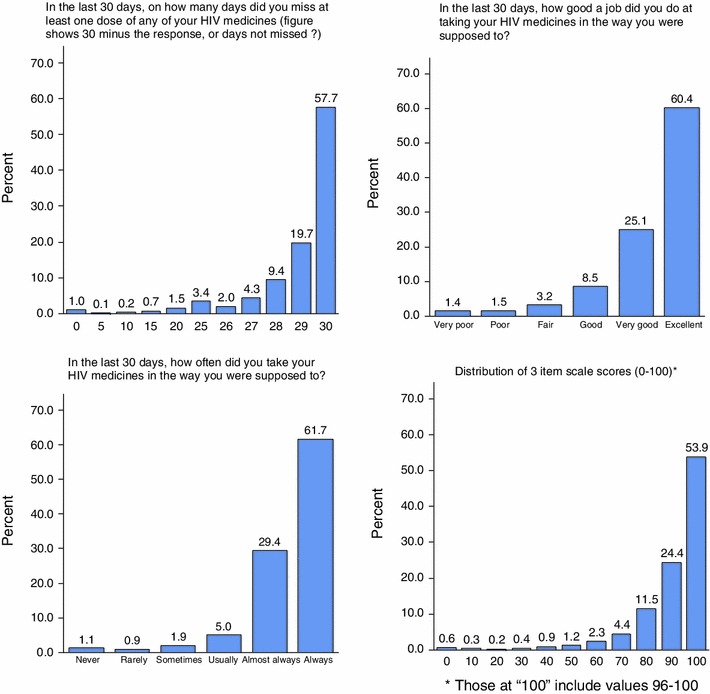
Distributions of the three items and the summary scale

Because the item and scale distributions were so similar, we combined them for purposes of illustrating their distributions (Fig. 1). All were skewed upward. The medians for the three items were all 100, and the median for the scale was 98.9 (data not shown). The percent scoring at the ceiling for the “days not missed,” “how good a job”, “how often,” and summary items were 58, 60, 62, and 54 % respectively. Those not scoring at the ceiling of the three-item scale used the full range of the rest of the 0–100 scale.

#### Internal Consistency Reliability

The Cronbach’s alphas for the three-item scale (Table 3) were quite high for all three samples (0.86–0.89).

## Discussion

There were three main findings from these analyses. First, the four rounds of cognitive testing allowed us to develop a set of items that we believe can be consistently understood by respondents from diverse sociodemographic and educational backgrounds. Second, the randomized experiment showed convincingly that the adherence scores did not differ between those asked about “all your HIV medications” and those who responded with a specific medication in mind. Third, in pilot testing the internal consistency reliability of three item scale was excellent.

We think results from cognitive interviewing can and should be more explicitly presented as a way of documenting the strengths and weaknesses of survey questions. Because in this case so many of the commonly used approaches to asking questions about medication taking proved unworkable in our cognitive testing, we thought that it would be useful to describe our findings—for example, the inconsistency with which patients understood concepts such as the “last week” or the “last month, the ambiguity of the term “as prescribed,” the confusion that visual analogue scales and percents created for many respondents, and the fact that patients were generally more comfortable using words than numbers in responses. While we tested these issues in two HIV care settings, we suspect that many of the findings would be generalizable to other populations being asked similar questions.

In previous work that used electronic drug monitoring as a reference we found that a rating scale performed better than other response sets [16] in correlating with the objective measure of adherence, and other investigators using the same rating item have since reported similar findings [17, 18]. We had previously speculated that the cognitive process of coming up with an adjectival rating appeared to correspond more closely to objective adherence data because it mapped more closely onto the cognitive process that patients used to form responses, which we thought was probably more an estimation process than an enumeration process. Our cognitive testing supports this theory. Only about half of respondents could demonstrate sufficient recall to describe details of their pill taking over a 30 days period. The majority were clearly estimating.

Interestingly, the rating item that we tested previously [16] was worded as follows: “Thinking back over the last month, on average how would you rate your ability to take all your [HIV] medications as prescribed.” (response options very poor to excellent) Even though the performance of this item has been excellent, our cognitive testing showed that respondents did not have a consistent understanding of either the “last month,” “rate your ability” or “as prescribed.” This led us to modify the question in ways that led to the current version, “In the last 30 days, how good a job did you do at taking your HIV medications in the way you were supposed to.”

There is a small literature that addresses the issue of whether adherence differs in clinically important ways among the individual medications that make up HIV antiretroviral regimens. Wilson et al. [19] using self-report measures from multiple individual antiretrovirals, concluded that patients tended to take (or not take) the individual antiretrovirals in their regimen as a group rather than taking some but not others at a given dosing time. McNabb et al. [20] used electronic drug monitoring pill caps (Aprex) and found very little differential adherence for different medications scheduled to be taken at the same time. Deschamps et al. [23] found little differential adherence using a self-report measure. Gardner et al. used pharmacy refill data and found that 15 % of patients in an unselected clinical population had “selective adherence,” defined as ≥5 % difference between two drugs in a regimen over an observation period of at least 60 days [21]. In a subsequent paper from a randomized trial Gardner et al. used self-report to assess differential adherence. Adherence was assessed separately for each component of the regimen, and patients were classified as having differential adherence if the assessments disagreed. In the 60 month trial with assessments every 4 months, 29 % reported differential adherence at least once, and 10 % reported it more than once [22].

While differential adherence clearly exists, and is probably clinical consequential [22], this research addresses a practical measurement question, not a clinical question. We tested the hypothesis that we would get quantitatively different results if we asked about patients’ global adherence with their HIV medications than if we asked them to respond with reference to a single, individual medication. In a web-based trial that had over 3,000 responses in each arm, the difference in the three-item scale was 0.8 points on a 100 scale (91.0 vs. 90.2). While this was significant at the level of *p* < 0.05, we do not think that this difference is clinically important.

We think the results of this web-based trial are useful because being able to ask respondents to report about their antiretroviral regimen overall is far simpler, and far less burdensome, than identifying individual medications and then asking questions about each one of them. The very small difference that we observed between arms was in the expected direction. Because we know that there is some differential adherence, we hypothesized that asking about non-adherence with three medications would be more sensitive than asking about non-adherence with a single medication. In short, we believe the data we present here supports the assertion that for most clinical and research applications it is reasonable to use these self-report items with reference to patients’ entire antiretroviral regimen. That said, for investigators interested specifically in differential adherence, these three items can be repeated for each of the pills in a regimen.

One of the primary challenges to measuring any socially desirable behavior by self-report is avoiding ceiling effects. That is, to the extent possible, one would like to avoid the problem of respondents over-reporting their adherence in such a way that all or most score at the top of the scale. This type of measurement error results in a measure with little useful variation to explore analytically. Of course, if the surveyed respondents were in fact highly adherent, then ceiling effects would be a function of the true underlying behavior rather than a type of measurement error. In this study, because we have no objective adherence measure with which to compare our adherence measures, we cannot identify the portion of our measurement that is error, and we cannot directly compare our findings with other studies that use related items in different populations [16–18]. However, in very recent reports, full or excellent adherence with current antiretroviral regimens has been reported in many settings to be quite high [28, 29].

This study has several limitations. First, it was not possible to do cognitive testing of all of the different items and response scales that have been used for self-report of medication adherence. We used judgment to select which items to test, and it is possible that other items or approaches would also have fared well in cognitive tests. Second, although we purposefully conducted our cognitive testing in a sociodemograpically diverse sample of persons with HIV, it is possible that testing in other populations would yield different results. Third, our web-based sample was largely well educated, gay, white men, and it is possible that the findings from the randomized experiment we conducted would have been different in a different population. Arguing against this is the fact that the distributional and psychometric characteristics from the clinic-based sample were strikingly similar to the web-based sample despite very different sample characteristics. Fourth, we cannot assess whether participants in the web-based survey did so fraudulently, as some have described [30]. Fifth, while we have presented findings from cognitive and psychometric testing, until we complete testing currently underway that includes an objective adherence measure as a comparator, we cannot make any statements about the validity of items or the scale. Finally, the use of these items in non-English speaking settings will require both careful translation and back translation [31, 32] and additional cognitive testing.

In conclusion, through detailed cognitive testing we have developed a new, short set of medication adherence self-report items. Next, in a large field test, we found that asking patients to report on adherence with their whole antiretroviral regimen produced similar results to asking them about individual medications. The three items and the resulting adherence scale had good distributional characteristics and an excellent Cronbach’s alpha. Both the lessons from our cognitive testing and the resulting items should be applicable to self-report of other medications used chronically for other conditions. Formal validity testing is underway, and rigorous testing of these items in a variety of other settings is encouraged.
